# MicroRNA‐488 inhibits proliferation and glycolysis in human prostate cancer cells by regulating PFKFB3

**DOI:** 10.1002/2211-5463.12718

**Published:** 2019-08-22

**Authors:** Jun Wang, Xiaojuan Li, Zhaoming Xiao, Yu Wang, Yuefu Han, Jun Li, Weian Zhu, Qu Leng, Yuehui Wen, Xinqiao Wen

**Affiliations:** ^1^ Department of Urology The Third Affiliated Hospital Sun Yat‐sen University Guangzhou China; ^2^ Department of Urology Fudan University Shanghai Cancer Center China; ^3^ Department of Oncology Shanghai Medical College Fudan University Shanghai China; ^4^ Center of Health Management Shenzhen Hospital Southern Medical University Shenzhen China; ^5^ Department of Urology Nanfang Hospital of Southern Medical University Guangzhou China; ^6^ Department of Urology Yue Bei People's Hospital Shaoguan China; ^7^ Department of Urology Shenzhen Hospital Southern Medical University Shenzhen China

**Keywords:** glycolysis, miR‐488, PFKFB3, proliferation, prostate cancer cell

## Abstract

Prostate cancer (PCa) remains the second leading cause of cancer‐related death among men in the United States, and its molecular mechanism remains to be elucidated. Recent studies have suggested that microRNAs may play an important role in cancer development and progression. By analyzing the Gene Expression Omnibus dataset, we found lower expression for miR‐488 in PCa than in normal tissues. Moreover, CCK‐8, EdU, glucose uptake, and lactate secrete assays revealed that overexpression of miR‐488 in PCa cell lines PC3 and DU145 resulted in inhibition of proliferation and glycolysis. In contrast, downregulation of miR‐488 expression promoted proliferation and glycolysis in PCa cells. Using a bioinformatic approach and dual‐luciferase reporter assays, we identified 6‐phosphofructo‐2‐kinase/fructose‐2,6‐bisphosphatase, isoform3 (PFKFB3), as a direct target of miR‐488. Inhibition of PFKFB3 also suppressed PCa cell glycolysis and proliferation. Our study suggests that miR‐488 inhibits PCa cell proliferation and glycolysis by targeting PFKFB3, and thus, miR‐488 may be a novel therapeutic candidate for PCa.

**Abbreviations**
CRPC: castration‐resistant prostate cancer
miRNAs: microRNAs
NCBI: National Center of Biotechnology Information
PCa: prostate cancer
PFKFB3: 6‐phosphofructo‐2‐kinase/fructose‐2,6‐bisphosphatase, isoform3

Prostate cancer (PCa) is a major health problem in older men and the second leading cause of cancer‐related death in males in the United States 1. Due to important advances in early screening and cancer management regimens, the overall survival rate and 5‐year survival rate of PCa patients have improved over the past 40 years 2. However, prognosis is greatly worsened once the disease progresses to castration‐resistant PCa (CRPC). Therefore, further elucidation of the molecular mechanism of PCa progression is of significant importance for finding effective treatment for CRPC.

MicroRNAs (miRNAs) are a class of noncoding small RNAs of approximately 20–22 nucleotides in length that degrade target mRNA or inhibit its translation through binding to the 3′‐UTR and thus play an important role in regulating gene expression 3. miRNAs are involved in the occurrence, proliferation, invasion, and metastasis of various cancers, such as PCa, glioma, ovarian cancer, and lung cancer. In addition, recent studies have demonstrated that miRNAs regulate the functions of various PCa cells during PCa progression 4, 5, 6, 7, and the relationship between PCa and miRNAs has been gradually dissected. However, as miRNAs are widely involved in various cellular functions of PCa, it is necessary to further study miRNAs and clarify their functions 4, 8, 9, 10.

miR‐488 is a miRNA that acts as a tumor suppressor or oncogene in various tumors, such as breast, lung, kidney, and liver cancers. Upregulation of miR‐488 in cells of these tumors may result in inhibition of target gene expression 11, 12, 13, 14, though the function and role of miR‐488 in PCa have not been fully elucidated. Our study found that miR‐488 was underexpressed in PCa and that it regulated PCa proliferation. Previous studies have revealed that the proliferation of tumor cells is related to the anaerobic glycolysis pathway, which is involved in many different processes, such as cell growth in an acidic microenvironment, matrix protein lysis, tumor angiogenesis, and energy production for DNA synthesis 15. We further experimentally confirmed that miR‐488 directly binds to the 3′‐UTR of the 6‐phosphofructo‐2‐kinase/fructose‐2,6‐bisphosphatase 3 (PFKFB3) gene, thereby regulating tumor proliferation and glycolysis in PCa. PFKFB3 is an isoenzyme that lacks phosphatase activity and promotes glycolysis 16; it is highly expressed in various cancer cells. miR‐488 suppresses glycolysis and then inhibits tumor proliferation through downregulation of PFKFB3 expression. This study investigated the expression level and role of miR‐488 in PCa and revealed its potential clinical application value.

## Materials and methods

### Expression of miR‐488 in prostate cancer

The GSE60117 dataset 17, which includes 56 cases of prostate tumors and 21 cases of normal prostate tissues, from the National Center of Biotechnology Information (NCBI) Gene Expression Omnibus database ( http://www.ncbi.nlm.nih.gov/geo/) was used to investigate expression of miR‐488 in PCa.

### Cell culture

Prostate cancer cell lines PC3, DU145, LNCap, 22RV1, and the normal prostate epithelial cell line RWPE‐1 were purchased from American Type Culture Collection. Cells were cultured in RPMI‐1640 (Gibco, Waltham, MA, USA) supplemented with 10% FBS or defined‐KSFM (Thermo Fisher Scientific, Waltham, MA, USA) in a humidified atmosphere of 5% CO_2_ at 37 °C.

### Total RNA extraction and quantitative real‐time PCR analysis

Total RNA was extracted using Trizol Reagent (TaKaRa, Kusatsu, Shiga, Japan), and quantitative real‐time PCR was performed with SYBR® Premix Ex Taq™ (TaKaRa) according to the manufacturer's instructions. The mRNA expression level of miR‐488 both in PCa and in normal prostate cells was measured using a Hairpin‐it™ miRNA qPCR Quantitation Kit (GenePharma, Shanghai, China) after reverse transcription. The expression level of miR‐488 was normalized to U6, whereas the expression level of PFKFB3 mRNA was normalized to β‐actin. The PCR primer sequences were as follows 5′‐3′: miR‐488, TGCGGCTTGAAAGGCTATT and ATGGAGCCTGGGACGAGAC; PFKFB3, GACGCACCCTTCCTGTCCTTTG and ACAAAGCCGCTGCACACACAA. The relative expression quantity was calculated using the $2^{-\Delta \Delta C_{t}}$ method. Each sample was replicated three times.

### Cell transfection

PC3 and DU145 cells were transfected with miR‐488 mimic, miR‐488 inhibitor, and negative control (RiboBio, Guangzhou, Guangdong, China) using Lipofectamine 3000 reagent (Invitrogen, Waltham, MA, USA) according to the manufacturer's instructions. Cells were harvested for further analysis at 24 h after transfection.

### Cell proliferation, migration, invasion, and apoptosis assays

Transfected cells were plated in 96‐well plates, and each well included 2 × 10^3^ cells. Subsequently, 10 μL CCK8 solution was added to each well, and the cells were cultured for 4 h. A microplate reader was used to detect the absorbance value of each well at a wavelength of 450 nm at the same time at 1, 2, 3, 4, and 5 days after plating. Each sample was replicated three times. For migration and invasion assays, a Transwell experiment was performed with or without 24 μg·μL^−1^ Matrigel. Briefly, transfected cells (2 × 10^4^) were plated in the upper Transwell chamber in 200 μL of serum‐free culture medium, and 10% serum culture medium was added to the bottom for 12 h of incubation. After incubation, the cells on the upper surface were removed, and those on the lower surface were stained with hematoxylin and eosin. Detection was performed by flow cytometry as described previously.

### EdU assay

A total of 1 × 10^5^ transfected cells were plated in each well of a confocal Petri dish, and 24 h later, EdU (Invitrogen) was added to the medium at a final concentration of 50 μm for 2 h. The cells were then fixed and treated with Apollo and Hoechst for nuclear staining and then mounted in standard mounting media. The stained cells were examined with a Nikon Eclipse E600 fluorescence microscope (Nikon Corporation, Konan Minato‐ku, Tokyo, Japan) and photographed with a Retiga 1300 Q‐imaging camera. The experiment was performed in triplicate.

### Detection of glucose uptake and lactate secretion levels

The intracellular glucose uptake rate and extracellular lactate secretion rate in PC3 and DU145 cells were detected using a Glucose Colorimetric Assay Kit (BioVision, Milpitas, CA, USA) and Lactate Assay Kit (BioVision) according to the manufacturer's instructions. Intracellular glucose uptake was detected using a standard glucose calibration curve performed under the same conditions. The extracellular lactate level was detected using a standard lactate calibration curve performed under the same conditions. Each sample was replicated three times.

### Plasmid construction and dual‐luciferase reporter assay

TargetScan was used to identify potential miR‐488 targets, and the predicted target gene associated with tumor glycolysis was selected based on the function of the encoded protein. The PFKFB3 3 ‐UTR contains a predicted binding site for hsa‐miR‐488; this and a fragment containing a mutated binding site sequence were synthesized and cloned into the NheI/SalI sites of the psiCHECK‐dual‐luciferase reporter vector. Annealing was performed as follows: 95 °C for 5 min and room temperature for 2 h. The reconstructed plasmids, named psiCHECK‐PFKFB3‐3′UTR‐WT or psiCHECK‐PFKFB3‐3′UTR‐MUT, were confirmed by restriction endonuclease digestion and sequencing. HEK293T cells were cultured in 24‐well plates, and the wild‐type or mutated PFKFB3 3′‐UTR sequence was cotransfected with miR‐488 mimic and negative control using Lipofectamine 3000. Luciferase activity was measured after 48 h of transfection using the dual‐luciferase reporter assay system according to the manufacturer's instructions.

### Western blotting analysis

Total protein was extracted from cells using RIPA lysis buffer (TaKaRa) according to the manufacturer's instructions, and the protein concentration was quantified using a bicinchoninic acid protein assay kit. Proteins were separated by 10% SDS/PAGE and then transferred onto a polyvinylidene fluoride membrane. The membrane was incubated with blocking buffer for 2 h at room temperature and then with the primary antibody anti‐PFKFB3 (Abcam, Cambridge, MA, USA) and Blotto overnight at 4 °C. Finally, the protein was visualized using ECL plus western blotting detection reagents (Biosciences, San Jose, CA, USA) and detected with an enhanced chemiluminescence kit.

### Statistical analysis

Statistical analysis was conducted using graphpad prism 5.0 software (GraphPad, San Diego, CA, USA). All measurement data are shown as the mean ± standard deviation (SD). Differences in measurement data between groups were analyzed using a two‐tailed Student's *t*‐test. Statistical significance was considered at *P *<* *0.05 for all analyses.

## Results

### The miR‐488 expression level is diminished in prostate cancer

**Figure 1:**
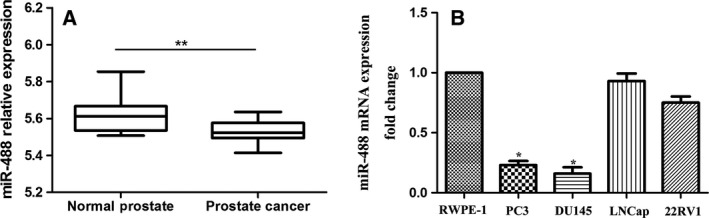
The expression level of miR‐488 in PCa tissues and cells. (A) Difference in expression of miR‐488 between PCa tissues and normal prostate tissues. (A) Expression of miR‐488 between PCa tissues and normal prostate tissues. (B) Expression of miR‐488 in PCa cell lines (including PC3, DU145, LNCaP, and 22RV1) and normal prostate epithelial cells (RWPE‐1) (*n* = 3). Error bars represent SD. Comparisons between groups were analyzed using *t*‐tests (two‐sided). Differences with *P* values of less than 0.05 are considered significant. * *P* < 0.05. ** *P* < 0.01.

To investigate the potential role of miR‐488 in PCa, we first downloaded the published GSE60117 dataset. As shown in Fig. 1A, expression of miR‐488 was significantly reduced in PCa tissues compared with normal prostate tissues. Subsequently, we detected the expression level of miR‐488 in several PCa cell lines using qRT–PCR. This analysis showed that the expression level of miR‐488 in PC3 and DU145 cells was significantly lower than that in normal prostate epithelial RWPE‐1 cells (Fig. 1B). Thus, PC3 and DU145 cells were chosen for further study.

### miR‐488 regulates proliferation and glycolysis in PC3 and DU145 cells

**Figure 2:**
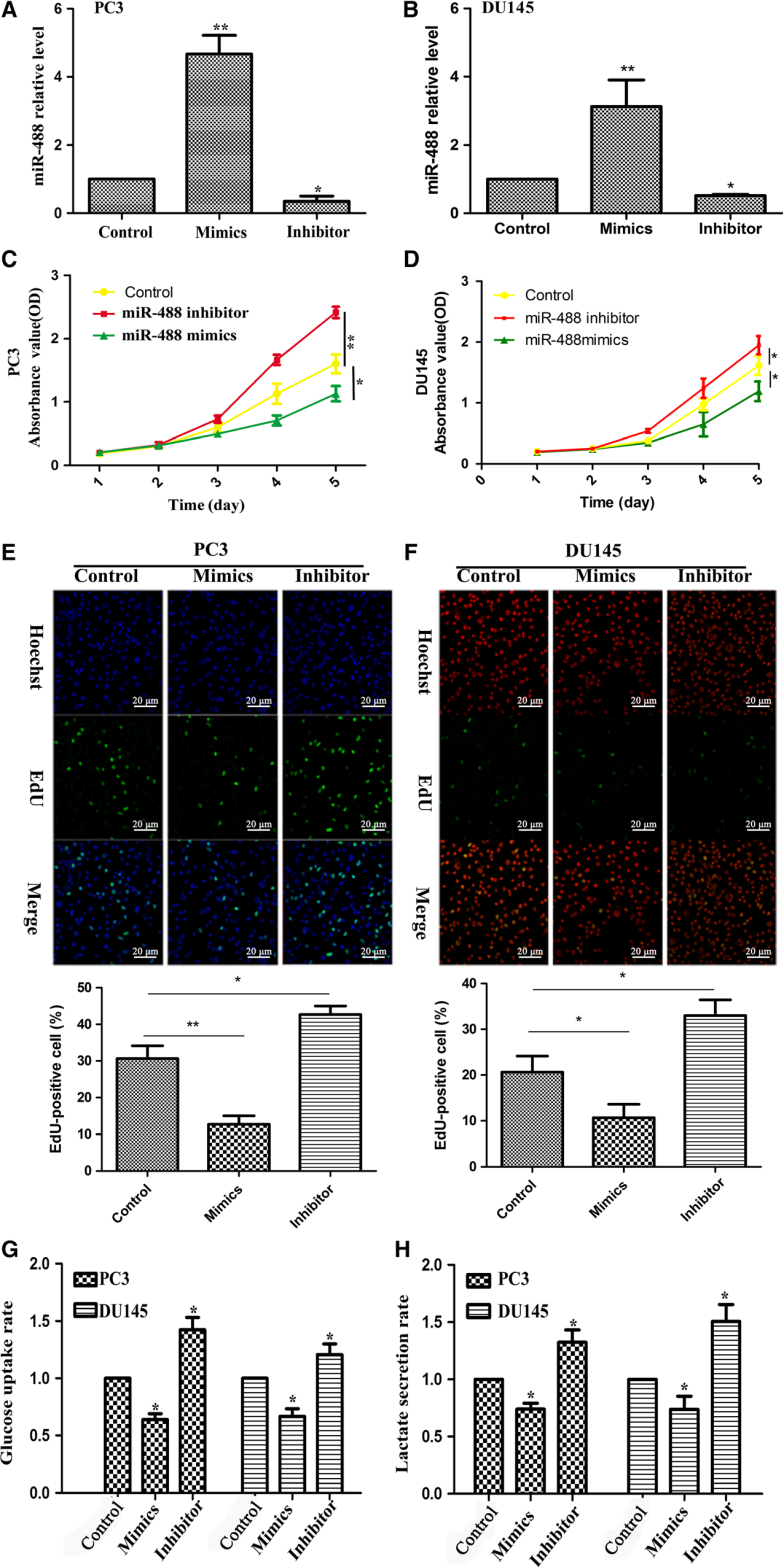
The function of miR‐488 in PCa cells. Expression of miR‐488 in PC3 cells (A) and DU145 cells (B) after transfection with miR‐488 mimic or inhibitor. The effect of miR‐488 on PC3 cell (C, E) and DU145 cell (D, F) proliferation was evaluated by the CCK‐8 assay (*n* = 3) and EdU labeling (*n* = 3). Original magnification was 200×. The role of miR‐488 in regulating glucose uptake (*n* = 3) (G) and lactate secretion (*n* = 3) (H) in PC3 and DU145 cells. Error bars represent SD. Comparisons between groups were analyzed using *t*‐tests (two‐sided). Differences with *P* values of less than 0.05 are considered significant. * *P* < 0.05. ** *P* < 0.01. Scale bar: 20 μm.

Next, we detected the expression level of miR‐488 in PC3 and DU145 cells after transfection with miR‐488 mimic, miR‐488 inhibitor or negative control. The results showed that the expression level of miR‐488 was significantly increased in the miR‐488 mimic‐transfected group but that it was decreased in the miR‐488 inhibitor‐transfected group in PC3 cells (Fig. 2A) and DU145 cells (Fig. 2B). To investigate the potential function of miR‐488, we transfected miR‐488 mimic or inhibitor into PC3 and DU145 cells and then assessed cell proliferation using a CCK‐8 assay and EdU labeling. The growth rate of miR‐488 overexpressing cells was significantly decreased compared to the negative control group. In contrast, after downregulation of miR‐488 by an inhibitor, the growth rate of cells was dramatically increased compared to the negative control group for both PC3 cells (Fig. 2C,E) and DU145 cells (Fig. 2D,F). To further evaluate the role of miR‐488 in regulating glycolysis, we transfected miR‐488 mimic or inhibitor into PC3 and DU145 cells. Compared with the negative control, a significant decrease in glucose uptake (Fig. 2G) and lactate secretion (Fig. 2H) was observed after miR‐488 mimic transfection, whereas it increased after miR‐488 inhibitor transfection. No significant differences in cell invasion, migration ability, or apoptosis were found after transfection with miR‐488 mimic or inhibitor (Fig. S1).

### miR‐488 directly targets PFKFB3 and modulates PFKFB3 expression

**Figure 3:**
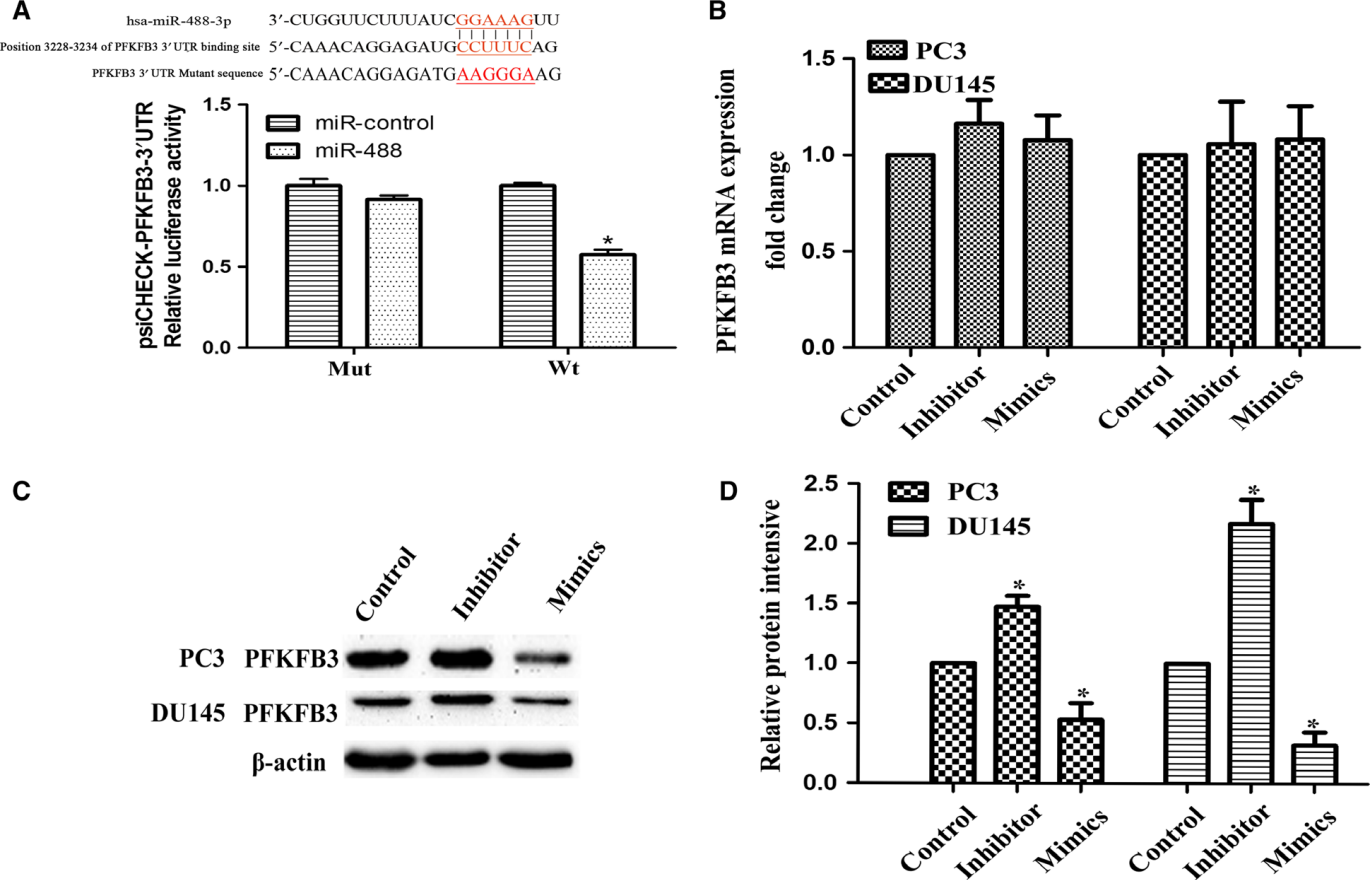
The relationship between miR‐488 and PFKFB3. (A) Luciferase activities were detected among groups of psiCHECK‐Wt and psiCHECK‐Mut cells cotransfected with miR‐488 mimic or miR‐control (*n* = 3). (B) Expression of PFKFB3 mRNA in PC3 and DU145 cells was detected by qRT–PCR after transfection with miR‐488 mimic or miR‐488 inhibitor (*n* = 3). (C, D) Expression of PFKFB3 protein in PC3 and DU145 cells was detected by western blotting after transfection with miR‐488 mimic or miR‐488 inhibitor (*n* = 3). Error bars represent SD. Comparisons between groups were analyzed using *t*‐tests (two‐sided). Differences with *P* values of less than 0.05 are considered significant. * *P* < 0.05.

According to the results of bioinformatics analysis, there is a putative binding site for miR‐488 in the 3′‐UTR of PFKFB3 (Fig. 3A). To validate whether PFKFB3 is a direct target of miR‐488, miR‐488 mimic or miR‐control and luciferase plasmids containing psiCHECK‐PFKFB3 3′‐UTR (Wt) or PFKFB3 3 3′‐UTR mutant (Mut) were cotransfected into HEK293T cells. Luciferase activity in HEK293T cells transfected with miR‐488 mimic and psiCHECK‐Wt was significantly decreased, whereas cotransfection with miR‐488 mimic did not markedly alter psiCHECK‐Mut reporter activity (Fig. 3A). Moreover, we examined the direct effect of miR‐488 on PFKFB3 expression in PC3 and DU145 cells, and the results indicated that overexpression of miR‐488 did not alter mRNA expression of PFKFB3 (Fig. 3B) but did significantly reduce PFKFB3 protein expression in PC3 and DU145 cells (Fig. 3C,D). Overall, these results show that miR‐488 negatively modulates PFKFB3 protein expression by directly binding to the 3′‐UTR of PFKFB3 mRNA.

### Inhibition of PFKFB3 suppresses proliferation and glycolysis in PC3 and DU145 cells

**Figure 4:**
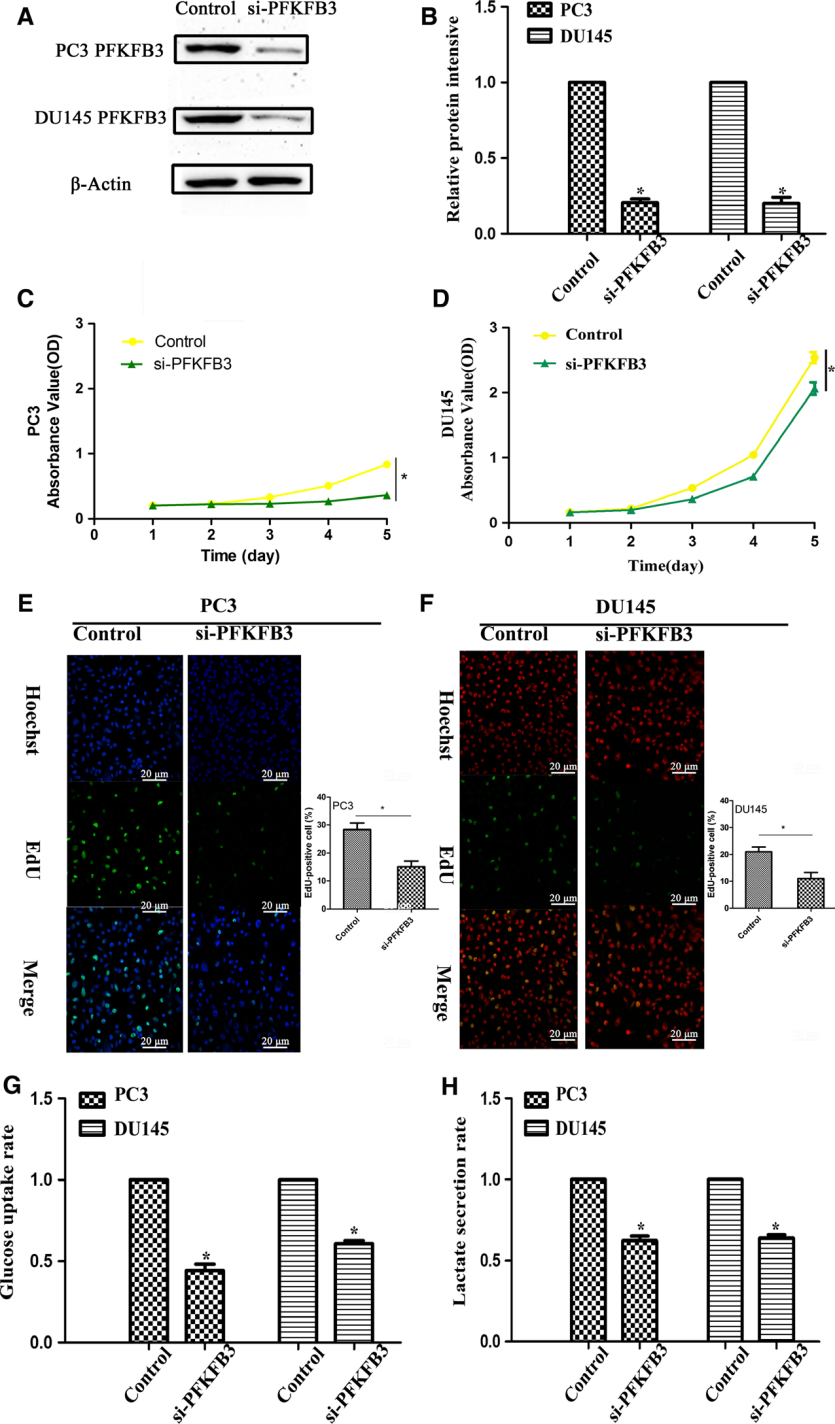
Inhibition of PFKFB3 suppresses proliferation and glycolysis in PCa cells. PCa cells PC3 and DU145 were transfected with siRNA targeting PFKFB3, and expression was measured by western blotting (*n* = 3) (A) and qRT–PCR (*n* = 3) (B). CCK‐8 (*n* = 3) (C, D) and EdU (*n* = 3) (E‐F) assays were performed to evaluate the proliferation of PCa cells. Glycolysis in PCa cells was detected by glucose uptake (G) and lactate secretion (H) assays. Error bars represent SD. Comparisons between groups were analyzed using *t*‐tests (two‐sided). Differences with *P* values of less than 0.05 are considered significant. *P < 0.05. Scale bar: 20 μm.

To further investigate whether miR‐488 inhibits proliferation and glycolysis of PC3 and DU145 cells by targeting PFKFB3, we used siRNA targeting PFKFB3 to downregulate its expression in PC3 and DU145 cells (Fig. 4A,B). We found that inhibition of PFKFB3 suppresses proliferation and glycolysis in both cell lines (Fig. 4C–H).

### PFKFB3 repression is required for miR‐488 to suppress proliferation and regulate glycolysis in PC3 and DU145 cells

**Figure 5:**
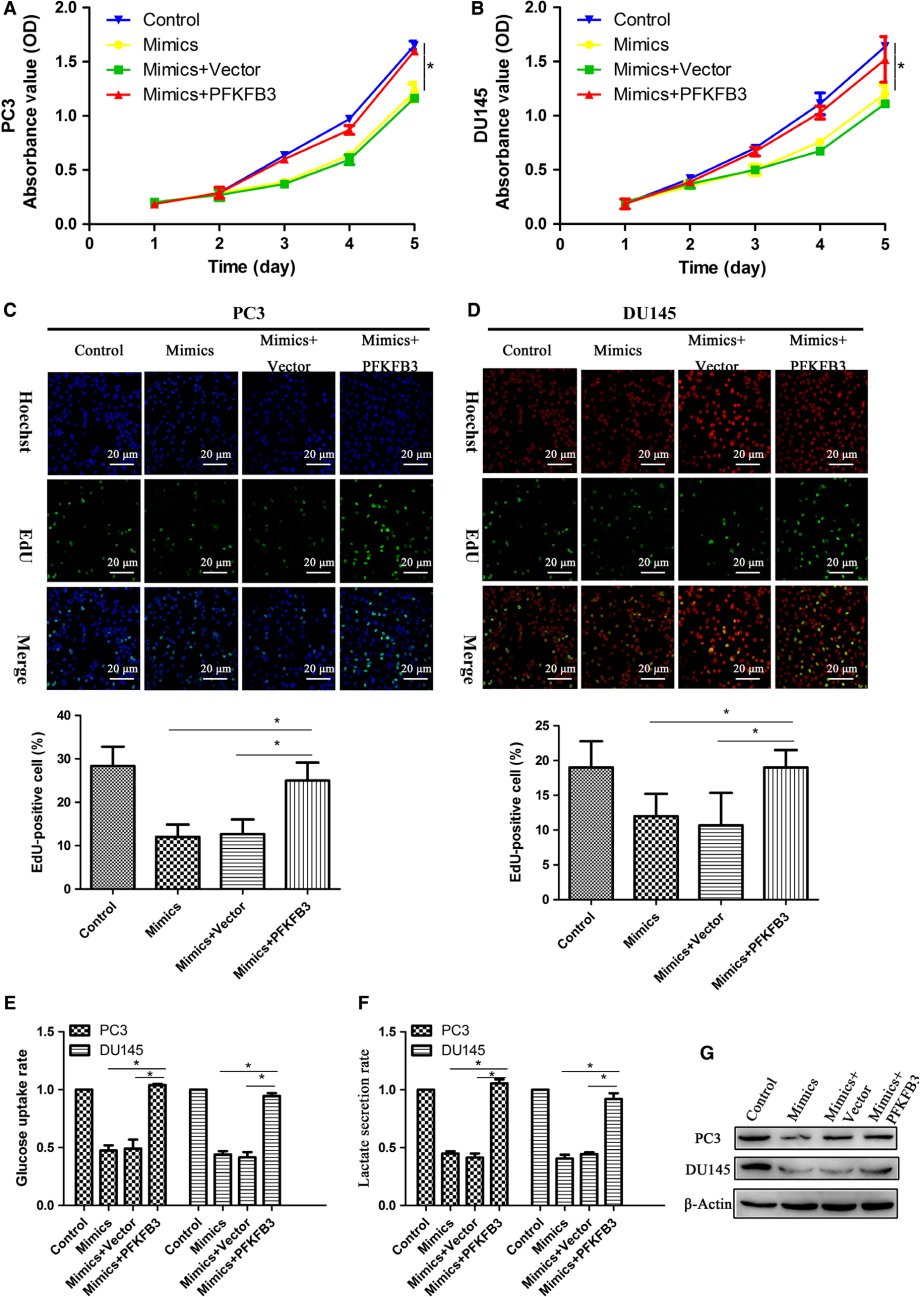
The function of miR‐488 regulates the proliferation of PCa cells by modulating PFKFB3. Proliferation of PC3 and DU145 cells transfected with the control (vector), miR‐488 mimics, or PFKFB3 overexpression plasmid was evaluated by CCK‐8 (*n* = 3) (A, B) and EdU labeling (C‐D) assays (*n* = 3). Glycolysis in PC3 and DU145 cells after transfection with the control (vector), miR‐488 mimics, or PFKFB3 overexpression plasmid was evaluated by glucose uptake (*n* = 3) (E) and lactate secretion (*n* = 3) (F) assays. Expression of PFKFB3 protein after transfection with the control (vector), miR‐488 mimics, or PFKFB3 overexpression plasmid in PC3 and DU145 cell lines (*n* = 3) (G). Error bars represent SD. Comparisons between groups were analyzed using *t*‐tests (two‐sided). Differences with *P* values of less than 0.05 are considered significant. *P < 0.05. Scale bar: 20 μm.

We have demonstrated that PFKFB3 mRNA is a target of miR‐488. To examine the potential role of miR‐488 and PFKFB3 in the proliferation of PCa cells, PFKFB3 was overexpressed in PC3 and DU145 cells. In both cell lines, the inhibition of proliferation (Fig. 5A–D) and glycolysis (Fig. 5E,F) caused by the miR‐488 mimic was rescued with transfection of the PFKFB3 plasmid. The decreased expression of PFKFB3 caused by miR‐488 mimic was also rescued by simultaneously transfect with miR‐488 mimics and PFKFB3 plasmid (Fig. 5G).

## Discussion

In recent years, an increasing number of studies have reported that miRNAs play an important role in regulating gene expression through mRNA degradation or translational inhibition 18, 19, 20. There is evidence that miRNAs are key regulatory factors in PCa and have the potential to be used as biomarkers for the diagnosis and prognosis of this cancer 21. In this study, we first analyzed the expression levels of miR‐488 in PCa tissues and normal tissues using a comprehensive NCBI gene expression database. The results showed that the expression levels of miR‐488 in PCa tissues were significantly lower than those in normal prostate tissues. Compared with normal prostate epithelial cells, the expression levels of miR‐488 in PC3 and DU145PCa cell lines were significantly reduced, and these cell lines were selected for further study. Furthermore, we found that miR‐488 inhibited the proliferation of and glycolysis in PC3 and DU145 cells through direct association with the 3′‐UTR of PFKFB3. These results indicate that miR‐488 plays an important role in the development of PCa.

PFKFB3 is one of four genes encoding 6‐phosphofructokinase‐2/fructose double phosphatase‐2, important regulatory enzymes of glycolysis, and fructose 2,6‐bisphosphate is a strong allosteric activator of phosphofructokinase‐1 22. The enzyme, which converts glucose‐6‐phosphate into glucose‐3‐phosphate, is present in a variety of tumor cell lines and leads to more glucose being metabolized through the glycolysis pathway to provide energy and synthetic substrates for tumor proliferation 23. PFKFB3 also plays an important role as a proto‐oncogene in the initiation and development of tumors. This study is the first to reveal the potential relationship between PFKFB3 and miR‐488 in PCa cell lines. In the process of exploring the mechanism of action of miR‐488, we used bioinformatics analysis to establish that PFKFB3 is a target gene of miR‐488, and we then confirmed that PFKFB3 is a direct target gene of miR‐488 using a dual‐luciferase gene reporter assay. These results indicate that miR‐488 downregulates expression of PFKFB3 by directly targeting the 3′‐UTR of its mRNA, thereby inhibiting the proliferation of PC3 and DU145 cells.

Previous studies have shown that a variety of miRNAs regulate the process of glycolysis in cells by targeting multiple key enzymes, also regulating the occurrence and development of tumors. However, unlike other tumors, glycolysis is not significantly upregulated at an early stage in PCa but is significantly upregulated when the tumor progresses to the castration‐resistant stage 24, 25, 26. miR‐488 directly participates in glycolytic regulation of PCa by regulating expression of PFKFB3, which suggests that miR‐488 may have a certain function in the transformation of PCa to the castration‐resistant stage. Consequently, miR‐488 may be a potential therapeutic target for CRPC.

In summary, our *in vitro* study demonstrates that miR‐488 regulates the proliferation of and glycolysis in PCa through targeted regulation of PFKFB3 expression. Upregulation of miR‐488 expression can inhibit PCa progression, and therefore, mir‐488 may be a potential target for PCa treatment.

## Conflict of interest

The authors declare no conflict of interest.

## Author contributions

JW and XL performed all experiments. ZX and YW collected and/or analyzed data. YH, JL, WZ, and QL repeated all experiments and interpreted the data. JW, XL, and YW contributed to writing the manuscript. XW contributed to the concept and design of the study. All of the authors approved the final manuscript.

## Supporting information


**Fig. S1.** The effect of miR‐488 on invasion, migration and apoptosis of prostate cancer cells**.** PC3 cells were transfected with miR‐488 mimic or inhibitor, and apoptosis was measured by propidium iodide (PI) and FITC‐Annexin V fluorescence (A). Cell invasion and migration were detected by Transwell assays (B). Scale bar: 100 μm.
