# Supplementary figures and images for: MicroRNA‐488 inhibits proliferation and glycolysis in human prostate cancer cells by regulating PFKFB3
Source: FEBS Open Bio. 2019 Aug 22;9(10):1798–807. doi: 10.1002/2211-5463.12718 (PMC6768114; doi:10.1002/2211-5463.12718)

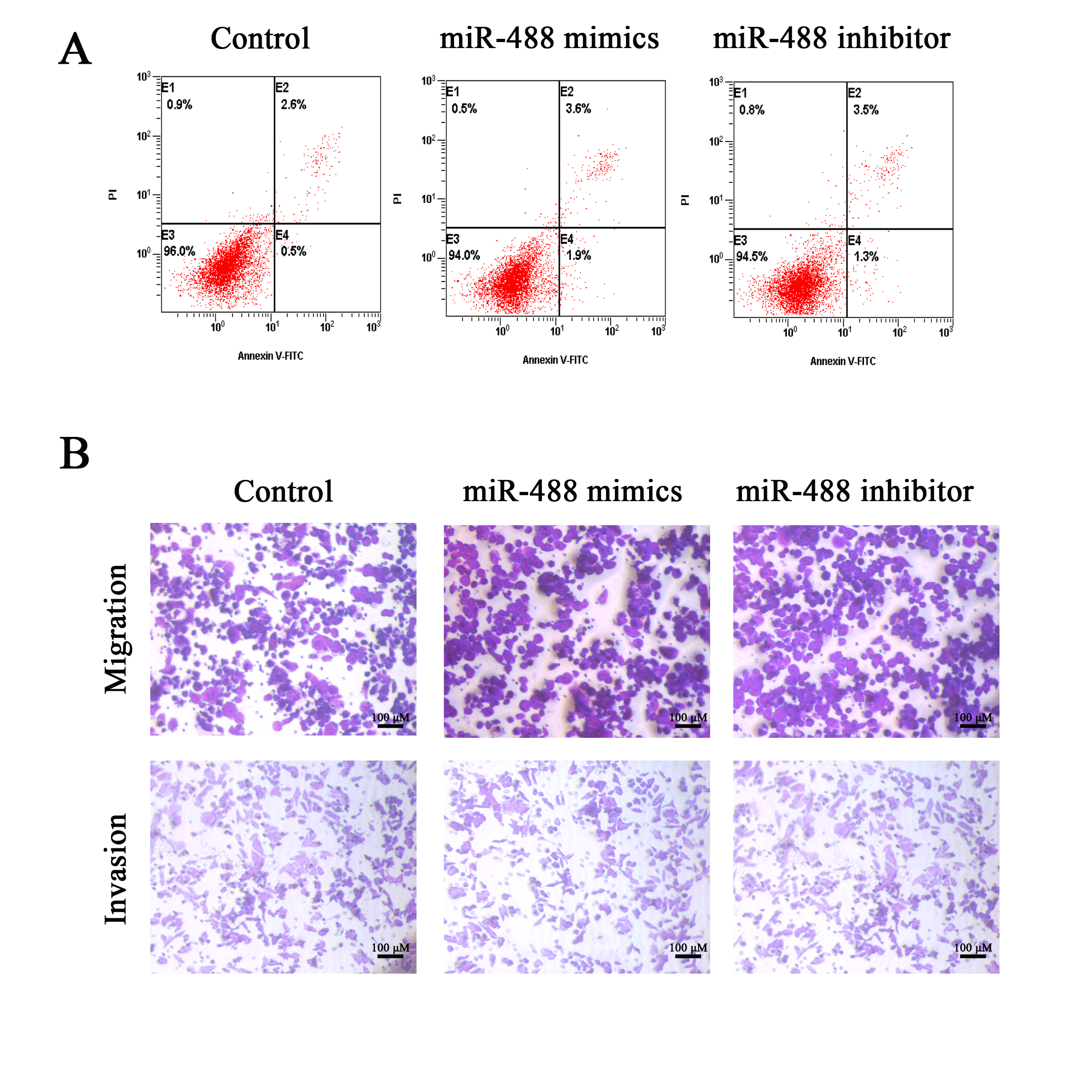

Supplement: Supplementary file 1 — Fig. S1. The effect of miR‐488 on invasion, migration and apoptosis of prostate cancer cells. PC3 cells were transfected with miR‐488 mimic or inhibitor, and apoptosis was measured by propidium iodide (PI) and FITC‐Annexin V fluorescence (A). Cell invasion and migration were detected by Transwell assays (B). Scale bar: 100 μm. [file FEB4-9-1798-s001.tif]
